# Non-Linear Relationship between Anti-Apolipoprotein A-1 IgGs and Cardiovascular Outcomes in Patients with Acute Coronary Syndromes

**DOI:** 10.3390/jcm8071002

**Published:** 2019-07-09

**Authors:** Nicolas Vuilleumier, Sabrina Pagano, Christophe Combescure, Baris Gencer, Julien Virzi, Lorenz Räber, David Carballo, Sebastian Carballo, David Nanchen, Nicolas Rodondi, Stephan Windecker, Stanley L. Hazen, Zeneng Wang, Xinmin S. Li, Arnold von Eckardstein, Christian M. Matter, Thomas F. Lüscher, Roland Klingenberg, Francois Mach

**Affiliations:** 1 Division of Laboratory Medicine, Diagnostic Department, Geneva University Hospital, 1205 Geneva, Switzerland; 2 Department of Internal Medicine specialities, Medical Faculty, Geneva University, 1205 Geneva, Switzerland; 3 Division of Clinical Epidemiology, University Hospital of Geneva, 1205 Geneva, Switzerland; 4 Center of Clinical Research, University of Geneva, 1205 Geneva, Switzerland; 5 Division of Cardiology, Cardiology Center, Geneva University Hospital, 1205 Geneva Switzerland; 6 Department of Cardiology, Bern University Hospital, University of Bern, 3010 Bern, Switzerland; 7 Center for Primary Care and Public Health (Unisanté), University of Lausanne, 1015 Lausanne, Switzerland; 8 Institute of Primary Health Care (BIHAM), University of Bern, 3010 Bern, Switzerland; 9 Department of General Internal Medicine, Inselspital, Bern University Hospital, University of Bern, 3010 Bern, Switzerland; 10 Department of Cardiovascular and Metabolic Sciences, Lerner Research Institute, Cleveland Clinic, Cleveland, OH 44195, USA; 11 Department of Cardiovascular Medicine, Heart and Vascular Institute, Cleveland Clinic, Cleveland, OH 44195, USA; 12 Institute of Clinical Chemistry, University Hospital of Zurich, 8091 Zürich, Switzerland; 13 Department of Cardiology, University Heart Center, University Hospital of Zurich, 8091 Zürich, Switzerland; 14 Center for Molecular Cardiology, University Heart Center, University Zurich, 8091 Zürich, Switzerland; 15 Heart Division, Royal Brompton and Harefield Hospital and Imperial College, Uxbridge UB9 6JH, London, UK

**Keywords:** acute coronary syndrome, biomarkers, anti-apolipoprotein A-I autoantibodies, GRACE score, C-statistics

## Abstract

Autoantibodies against apolipoprotein A-I (anti-apoA-I IgGs) are prevalent in atherosclerosis-related conditions. It remains elusive whether they improve the prognostic accuracy of the Global Registry of Acute Coronary Events (GRACE) score 2.0 (GS) in acute coronary syndromes (ACS). In this prospective multicenter registry, 1713 ACS patients were included and followed for 1 year. The primary endpoint (major adverse cardiovascular events (MACE)) was defined as the composite of myocardial infarction, stroke (including transient ischemic attack), or cardiovascular (CV) death with individual events independently adjudicated. Plasma levels of anti-apoA-I IgGs upon study inclusion were assessed using ELISA. The association between anti-apoA-I IgGs and incident MACE was assessed using Cox models with splines and C-statistics. One-year MACE incidence was 8.4% (144/1713). Anti-apoA-I IgG levels were associated with MACE with a non-linear relationship (*p* = 0.01), which remained unchanged after adjusting for the GS (*p* = 0.04). The hazard increased progressively across the two first anti-apoA-I IgG quartiles before decreasing thereafter. Anti-apoA-I IgGs marginally improved the prognostic accuracy of the GS (c-statistics increased from 0.68 to 0.70). In this multicenter study, anti-apoA-I IgGs were predictive of incident MACE in ACS independently of the GS but in a nonlinear manner. The practical implications of these findings remain to be defined.

## 1. Introduction

Over the last decades, the insight into the pathogenesis of atherosclerosis evolved from a lipid-centered disease to a predominantly T-helper (Th)-1 driven immune response against various antigens and auto-antigens within atherosclerotic plaques [1,2,3]. Among the latter, both cellular and humoral autoimmune responses against different membrane proteins, native or modified lipoproteins have been shown to modulate the course of atherogenesis either in a pro- or anti-atherogenic manner [4,5]. Reflecting this intrinsic biological complexity, several autoantibodies have been detected in sera of individuals and associated either with an increased or decreased cardiovascular (CV) risk [6,7,8,9,10].

Among autoantibodies targeting lipoproteins potentially associated with increased CV risk, those directed against apolipoprotein A-I (apoA-I), the major protein of high-density lipoprotein (HDL), appear as appealing candidates. Indeed, most observational studies performed so far indicate that antibodies against anti-apoA-I (anti-apoA-I IgGs) were an independent CV risk factor predictive of poor prognosis, both in the general population and in different CV risk settings, associated with a systemic pro-inflammatory profile and atherosclerotic plaque vulnerability [11,12,13,14,15,16,17,18,19,20,21,22]. Furthermore, in vitro and in vivo studies indicate that these autoantibodies can promote the loss of HDL anti-oxidative function [15], induce sterile inflammation, atherogenesis, myocardial necrosis, and death in experimental mouse models through activation of a specific innate immune receptor complex consisting of Toll-like receptor (TLR)2, 4, and CD14 [23,24,25].

Nevertheless, it is unknown whether anti-apoA-I IgGs provide incremental prognostic value over the Global Registry of Acute Coronary Events (GRACE) risk score 2.0 [26] after acute coronary syndromes (ACS), and whether such an association could follow a nonlinear relationship with the hazards, as recently shown for HDL-cholesterol regarding incident overall mortality [27]. Finally, we explored the associations between anti-apoA-I IgGs and other clinical and biological variables available for this cohort [28,29], such as lipid profile, high sensitivity C-reactive protein (hs-CRP), *N*-terminal pro-brain natriuretic peptide (NT-proBNP), high-sensitivity troponin T (hs-cTnT), trimethyl-amine-*N*-oxide (TMAO), red blood cells, white blood cells, and platelets counts.

## 2. Experimental Section

### 2.1. Study Population

These findings are derived from the prospective multicenter Swiss ACS Cohort [28,30,31], as part of the Special Program University Medicine (SPUM), which enrolled patients referred for coronary angiography with a primary diagnosis of ACS at one of the participating University Hospitals (Zurich, Bern, Lausanne, Geneva; Clinical Trials Registration number: NCT01000701; www.spum-acs.ch) between December 2009 and October 2012. Of 2168 included Biomarker Cohort 1 patients, 1713 ACS patients had available anti-apoA-I IgG measurements in addition to the previously described clinical and laboratory data and were included in this study.

Briefly, inclusion criteria were: (1) patients older than 18 years admitted within 5 days (preferably within 72 h) after pain onset with the main diagnosis of S–T segment (ST)-elevation myocardial infarction (STEMI), non-ST-elevation myocardial infarction (NSTEMI), or unstable angina (UA); (2) persistent ST-segment elevation or depression, T-inversion or dynamic Electrocardiogram (ECG) changes, or new left bundle branch block (LBBB); (3) evidence of positive troponin by local laboratory reference values with a rise and/or fall in serial troponin levels; (4) known coronary artery disease, specified as status after myocardial infarction (MI), coronary artery bypass graft (CABG), percutaneous coronary intervention, or newly documented ≥50% stenosis of an epicardial coronary artery during the initial catheterization. Exclusion criteria comprised of (1) severe physical disability; (2) inability to comprehend study; (3) less than 1 year of life expectancy for non-cardiac reasons. Patients were followed-up for 1 year. All subjects gave written informed consent according to the declaration of Helsinki, and the study was approved by the respective institutional review boards.

### 2.2. Definitions of Study Outcomes

The primary outcome of this study consisted of major adverse CV events (MACE), defined as a composite of myocardial infarction (MI), stroke (including transient ischemic attack), or CV death, as described previously [28,30,31]. Participants were first followed up at 1-year post-ACS by telephone by a trained study nurse to attend a clinical visit. If patients were unable to attend the clinic visit, follow-up was performed in the following order: (1) phone call, (2) postal mail or email, (3) through family members, or (4) via primary care physician or cardiologist. Reviews of medical records and clinical visits were conducted for the 1-year outcomes. Clinical endpoints were adjudicated by a panel of three independent certified senior cardiologists blinded to anti-apoA-1 IgG results using standardized pre-specified adjudication forms.

### 2.3. Biochemical Analyses

Blood was drawn from the arterial sheath at coronary angiography and centrifuged at 2700 × *g* for 10 min at room temperature to obtain serum, and then frozen and stored in aliquots at −80 °C. Routine laboratory tests (including lipid profile, hs-CRP, NT-proBNP, hs-cTnT, TMAO, red blood cells, white blood cells, and platelets count) were performed according to laboratories of each Institution, and the corresponding detailed analytical aspects have been reported earlier [28,29]. Estimated glomerular filtration rate (eGFR; in mL/min per 1.73 m^2^) was calculated for each cohort using the modification of Diet in Renal Disease study (MDRD) equation.

#### Anti-Apolipoprotein A-I IgG Levels

Anti-apoA-I IgGs were measured as previously described [11,12,13,16,20,21,22], using frozen EDTA plasma aliquots, stored at −80 °C until analyses. Maxisorp plates (Nunc^TM^, Roskilde, Denmark) were coated with purified, human-derived delipidated apolipoprotein A-I (20 μg/mL; 50 μL/well) for 1 h at 37 °C. After being washed, all wells were blocked for 1 h with 2% bovine serum albumin (BSA) in a phosphate buffer solution (PBS) at 37 °C. Participants’ samples were also added to a non-coated well to assess individual non-specific binding. After six washing cycles, a 50 μL/well of signal antibody (alkaline phosphatase-conjugated anti-human IgG; Sigma-Aldrich, St Louis, MO, USA), diluted 1:1000 in a PBS/BSA 2% solution, was added and incubated for 1 h at 37 °C. After washing six more times, phosphatase substrate *p*-nitrophenyl-phosphate-disodium (Sigma-Aldrich, St Louis, MO, USA) dissolved in a diethanolamine buffer (pH 9.8) was added and incubated for 20 min at 37 °C (Molecular Devices^TM^ Filter Max F3, Molecular Devices, San Jose, CA, USA). Optical density (OD) was determined at 405 nm, and each sample was tested in duplicate. Corresponding non-specific binding was subtracted from mean OD for each sample. The specificity of detection against lipid-free and unmodified apoA-I has been determined previously by conventional saturation tests, Western blot, and LC-MS analyses [12]. At an intermediate value of 0.6 OD, the interassay coefficient of variation was 9% (n = 5), and the intra-assay CV was 5% (n = 5).

### 2.4. Statistical Methods

The data were expressed as medians ± interquartile ranges for continuous variables and as numbers and percentages for categorical variables. The correlation between anti-apoA-I IgG and other biomarkers was evaluated by nonparametric approach (Spearman rank correlation). Given the fact that anti-apoA-I IgG seropositivity cut-off has not been defined nor validated on EDTA plasma, continuous anti-apoA-I IgG levels in OD were categorized according to quartiles and/or used as a continuous variable to assess the association with clinical outcomes. Time-to-first event or composite events were analyzed censoring patients at 365 days or last valid contact date. Univariate and adjusted (for GRACE score 2.0) associations of anti-apoA-I IgG categories with study endpoint was evaluated using Cox proportional hazards models and expressed with hazard ratios (HRs) and 95% CI. The predictive value of anti-apoA-I IgG over and above a reference model was assessed by Harrell’s C-statistics calculated from a Cox proportional hazards regression model based on a logistic model using the GRACE risk score as a reference [26]. To identify a possible nonlinear relationship between MACE risk and anti-ApoA-I IgG continuous values, we used a Cox regression model with splines (with a degree of freedom 2) [32]. A two-sided *p*-value <0.05 was considered statistically significant. Statistical analyses were performed using STATA software^®^ (Version 15, STATA Corp., College Station, TX, USA).

## 3. Results

### 3.1. Baseline Demographic and Biological Characteristics

Baseline characteristics of patients and pertinent data are summarized in Table 1. During the 1-year follow-up, 144 patients (8.4%) met the composite endpoint of MACE. Among them, 57 had a myocardial infarction, 26 had a stroke, and 68 died from CV causes.

### 3.2. Associations with Major Adverse Cardiovascular Events (MACE) at 1-Year of Follow-Up

Traditional CV risk factors, as well as the GRACE risk score 2.0, hs-CRP, hs-cTnT, NT-proBNP, TMAO levels, and anti-apoA-I IgG, upon study inclusion, were found to be higher in individuals with MACE at 1-year post-ACS than in those without (Table 1). As shown in Table 2 and Table 3, most of the traditional CV risk factors, history of MI, renal function, and conventional biomarkers were significantly associated with MACE at 1 year in univariate analyses. Of note, HDL cholesterol was not associated with the study endpoint. As previously reported in the SPUM-ACS cohort [33], lower low-density lipoprotein (LDL) and total cholesterol levels were significantly associated with a decreased risk of MACE.

**Figure 1:**
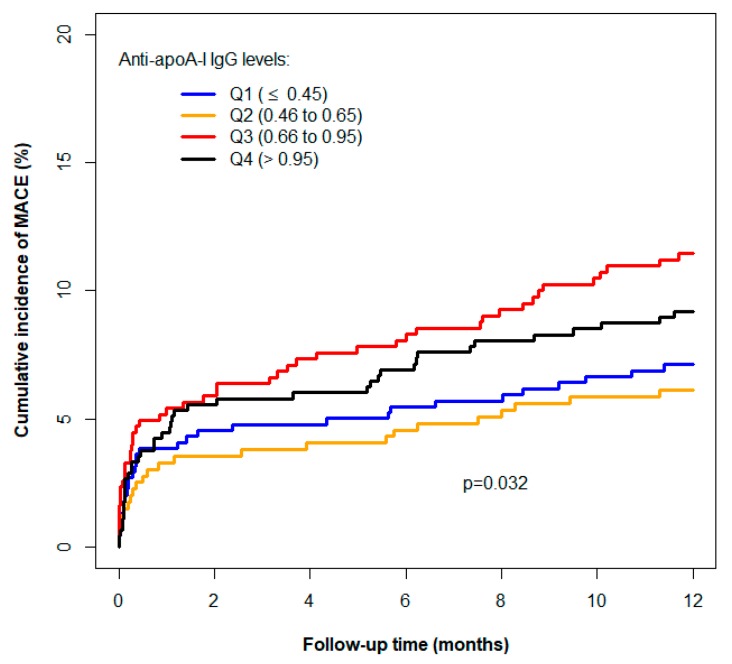
Cumulative incidence of major adverse cardiovascular events (MACE) (myocardial infarction, stroke, TIA, or cardiovascular (CV) death) according to autoantibodies against apolipoprotein A-I (anti-apoA-I IgG) optical density (OD) quartiles.

**Figure 2:**
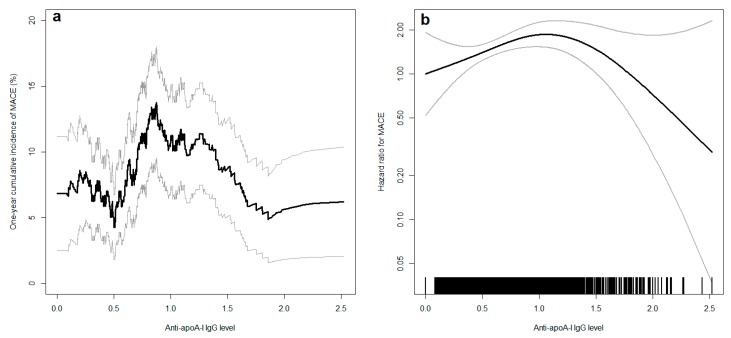
(**a**) Cumulative incidence of major adverse cardiovascular events (MACE) at one year according to autoantibodies against apolipoprotein A-I (anti-apoA-I IgG) levels (in optical density (OD)) in univariate analyses (black line). (**b**) Hazard ratio for MACE according to anti-apoA-I IgG levels (black line) adjusted for the categories of the Global Registry of Acute Coronary Events (GRACE) score 2.0. The vertical black lines above the x-axis represent the distribution of anti-apoA-I IgG levels in the study population: each vertical line represents the value of one participant. In both panels, the grey lines represent the 95% confidence interval. The distribution of anti-apoA-I IgG values was as followed: from 0 to 0.5 OD: n = 544 (31.8%); >0.5 and ≤1.0: n = 774 (45.2%); >1.0 and ≤1.5: n = 292 (17.0%); >1.5 and ≤2.0: n = 89 (5.2%); >2.0: n = 14 (0.8%).

Anti-apoA-I IgG levels were associated with MACE (Table 3). Cumulative incidence curves are represented in Figure 1 by anti-apoA-I IgGs quartiles. The adjustment for the GRACE score did not modify the association (Table 4). The relationship between the incidence of MACE and anti-apoA-I IgG levels was not linear since the cumulative incidence and the hazard ratio did not change progressively with the anti-apoA-I IgG level (Figure 1, Table 3). The non-adjusted relationship between the one-year risk of MACE and anti-apoA-I IgGs is depicted in more detail in Figure 2a. An inverted U-shaped reminiscent association was apparent for MACE, with a progressively increasing risk up to an anti-apoA-I IgG level of 1.0 OD, before an apparent decrease, difficult to interpret due to the broadness of the 95% confidence interval. In an additional Cox model accounting for this nonlinearity and adjusted for the GRACE score, the relation between the hazard and MACE remained identical (Figure 2b). With this model, the non-linearity of the relationship (*p* = 0.0110) and the association between MACE and anti-apoA-I IgG level (*p* = 0.0068) were statistically significant. Of note, anti-apoA-I IgG values spanning from 0 to 1.0 OD represented 77% of all anti-apoA-1 IgGs values, whereas values above 1.0 represented 23% of all anti-apoA-I IgG values on this cohort. Secondary analyses revealed that the main driver of anti-apoA-I IgG-related MACE risk in the composite first endpoint was the recurrence of MI as anti-apoA-I IgG levels were not predictive of stroke or CV deaths (Figure S1, Supplementary Material).

C-statistics confirmed that anti-apoA-I IgG levels were a modest but significant predictor for MACE. When added to the GRACE risk score for MACE prediction at one year, anti-apoA-I IgG marginally improved the prognostic accuracy of the model: C-statistics changed from 0.680 for Cox model with the categories of a GRACE risk score to 0.698 for Cox model combining the categories of GRACE risk score and anti-apoA-I IgG levels in a continuous way.

### 3.3. Anti-apoA-I IgG Associations in Acute Coronary Syndrome (ACS)

To further explore the associations between anti-apoA-I IgGs and patients’ clinical and biological characteristics in ACS, we analyzed the distribution of the latter according to anti-apoA-I IgG quartiles. As shown in Table 5 and Table 6, an inverse association was observed with the prevalence of diabetes that decreased among the increasing anti-apoA-I IgG categories, and a trend toward increased representation of valvular disease along anti-apoA-I IgG categories was noted. Furthermore, Spearman correlation coefficients showed modest but statistically significant positive associations between anti-apoA-I IgG levels and NT-proBNP and hemoglobin levels, while negative associations were retrieved between anti-apoA-I IgG and neutrophil, eosinophil, and platelet counts (Table S1, Supplementary Material). Close to significant associations (*p* ≤0.1) were noted with age, hs-cTnT, hemoglobin, hematocrit, leucocytes, creatinine, total cholesterol, and triglyceride levels. No other significant association was retrieved, notably with TMAO levels (Table S1). None of these anti-apoA-I IgG associations would have remained significant after the application of Bonferroni correction (significance threshold: 0.05/24: *p* = 0.002).

## 4. Discussion

The key finding of the present study is that anti-apoA-I IgG was predictive of incident MACE independently of the GRACE score 2.0, one of the strongest prognostic stratification tools currently validated in ACS. Furthermore, the prognostic accuracy of the GRACE score was slightly improved (+2%) by adding anti-apoA-I IgGs to it, according to C-statistics. Because of the important controversy regarding the reliability of C-statistics comparison and reclassification statistics (net reclassification index and integrated discrimination index) in embedded models due to the important false positive findings [34,35], we did not perform further statistical analyses to interpret this difference. Indeed, we feel that a 2% increase in C-statistics is unlikely to be clinically meaningful or to change current clinical practice in ACS. Nevertheless, these results strengthen the proof-of-principle that anti-apoA-I IgGs are predictive of a poor CV prognosis, as suggested earlier in the general population and in different high CV risk settings [11,12,13,14,15,16,17,20,21,22].

The second notable finding of this study is that the association between anti-apoA-I IgGs and MACE was not linear over the full range of anti-apoA-I IgG values. Indeed, the relationship was linear for the first 87% anti-apoA-I IgG values but started to decline for the remaining 23% highest anti-apoA-I IgG values. According to Cox regression models with splines confirming the significant nature of this non-linear relationship with MACE, we hypothesized that anti-apoA-I IgGs would mirror the U-shaped relationship between HDL-cholesterol and outcomes reported lately [27]. Nevertheless, there was no association between HDL-cholesterol and MACE or between HDL-cholesterol and anti-apoA-I IgGs in this study. Furthermore, due to the small number of events in this last 23% portion of anti-apoA-I IgG values, the confidence intervals of our regression model increased, preventing us from concluding on a possibly inverted U-shape relationship with MACE or the occurrence of a plateau at high anti-apoA-I IgG values. To the best of our knowledge, this is the first report of a non-linear association between anti-apoA-I IgG levels and MACE. Whether this non-linear association is specific for the ACS population is still elusive, but should be taken into account in future studies in order to avoid possible false negative findings related to the classic assumption of a proportional risk increase between anti-apoA-I IgG levels and hazards.

To further explore the possible associations between biological and clinical parameters with anti-apoA-I IgG levels in ACS, our linear regression analyses of anti-apoA-I IgG quartiles did not identify specific items markedly associated with anti-apoA-I IgG, and we did not reproduce the often reported association with previous MI history [11,20,21,22]. Interestingly, a trend was observed with the presence of valvular diseases, and an inverse relationship with the history of diabetes. This possible association with the presence of valvular diseases is in line with the concept supporting a causal role between humoral autoimmunity and different valvular diseases historically exemplified by rheumatic heart disease [6]. Nevertheless, as anti-apoA-I IgGs have never been shown to be associated with valvular diseases, these results open a new field of investigations, especially as we could not specify more in detail with which specific valvular disease these autoantibodies were associated. Furthermore, as anti-apoA-I IgGs were found to be higher in type 2 diabetic patients and associated with a CVD [17], the inverse association reported here between the prevalence of diabetes across anti-apoA-I IgG quartiles was unexpected and thus requires further confirmation. Of note, we did not retrieve any association between anti-apoA-I IgG and hs-CRP levels, despite previous reports, notably in the general population, where such association was retrieved [11]. Because anti-apoA-I IgG has been shown to promote various inflammatory cytokines production [10], including interleukin-6 regulating CRP liver production, this absence of association was unexpected. The reason for such difference is unknown and may be explained by differences related to the acute phase the samples of ACS patients were taken when compared to the stable situation of CoLaus individuals.

On the other hand, the inverse associations retrieved between these antibodies and lymphocyte and platelet counts are similar to what has been retrieved in HIV patients [36], and the possible causal nature of such associations is still under investigation. The inverse association between anti-apoA-I IgGs and eosinophil counts has not been reported. Whether patients with high anti-apoA-I IgG levels could be less prone to develop an allergic coronary syndrome (known as the Kounis syndrome) [37] should be further explored. The weak but significant positive anti-apoA-I IgG correlation with NT-proBNP, one of the strongest prognostic biomarkers of mitigated prognosis, may be related to the prognostic value previously ascribed to anti-apoA-I IgGs in different settings [11,12,13,14,15,16,17,20,21,22]. Because experimental evidence indicates that these autoantibodies could per se promote atherogenesis and atherothrombosis, as well as mitigate mouse survival by eliciting sterile inflammation through TLR2/4 and CD14 signaling [22,23,24,25], the present results reinforce the notion that anti-apoA-I IgGs could represent a future therapeutic target rather than a mere innocent bystander. Nevertheless, due to the non-linear relationship between these antibodies and MACE, knowing whether and how assessing anti-apoA-I IgG levels could affect current medical practice remains to be demonstrated.

The major limitation of this study relies on the fact that we did not assess other auto-antibodies of potential CV relevance, which is outside of the scope of the present work. The strength of the study relies on the fact that so far, it represents the largest and best characterized ACS cohort studying the prognostic value of anti-apoA-I IgGs in ACS.

## 5. Conclusions

In conclusion, anti-apoA-I IgGs are associated with incident MACE in ACS, independently of the GRACE 2.0 score, and with a nonlinear relationship to the hazards. Whether anti-apoA-I IgGs could provide incremental information over the existing risk stratification tool still needs to be determined.

## Figures and Tables

**Table 1:** Baseline characteristics.

Patient Characteristics	All Patients (n = 1713)	MACE * (n = 144)	No MACE * (n = 1569)
Age, years			
Median (IQR)	64 (54–74)	76 (64–81)	63 (53–73)
BMI, kg/m^2^			
Median (IQR)	26.5 (24.2–29.4)	26.0 (24.1–30.4)	26.5 (24.2–29.4)
Missing data	23	9	14
Female, n (%)	363/1713 (21.2%)	35/144 (24.3%)	328/1569 (20.9%)
Current smoker, n (%)	690/1680 (41.1%)	44/138 (31.9%)	646/1542 (41.9%)
Diabetes history, n (%)	310/1713 (18.1%)	47/144 (32.6%)	263/1569 (16.8%)
MI history, n (%)	246/1713 (14.4%)	31/144 (21.5%)	215/1569 (13.7%)
Hypertension history, n (%)	993/1713 (58.0%)	100/144 (69.4%)	893/1569 (56.9%)
Dyslipidemia history, n (%)	1031/1711 (60.3%)	82/143 (57.3%)	949/1568 (60.5%)
Valvular disease history, n (%)	33/1713 (1.9%)	10/144 (6.9%)	23/1569 (1.5%)
Statin use, n (%)	503/1702 (29.6%)	50/141 (35.5%)	453/1561 (29.0%)
Beta-blocker use, n (%)	410/1699 (24.1%)	54/141 (59.3%)	356/1558 (22.8%)
GRACE score 2.0			
Median (IQR)	123 (106–142)	144 (120–165)	121 (104–140)
Hs-CRP, mg/l			
Median (IQR)	3.0 (1.2–8.0)	5.8 (2.3–18.9)	2.7 (1.1–7.6)
Missing data	203	11	192
Hs-cTnT, pg/L			
Median (IQR)	0.22 (0.07–0.75)	0.51 (0.13–1.92)	0.21 (0.06–0.70)
Missing data	194	10	184
TMAO, µmol/L			
Median (IQR)	2.93 (1.99–4.97)	4.25 (2.12–7.35)	2.86 (1.98–4.85)
Missing data	360	38	322
Anti-ApoA-I IgGs, OD			
Median (IQR)	0.66 (0.44–0.97)	0.76 (0.49–0.98)	0.65 (0.44–0.97)
HDL, mmol/L			
Median (IQR)	1.13 (0.94–1.39)	1.15 (0.94–1.46)	1.12 (0.94–1.38)
missing data	65	8	57
LDL, mmol/L			
Ledian (IQR)	3.08 (2.34–3.84)	2.72 (2.04–3.45)	3.11 (2.39–3.86)
Missing data	68	8	60
Total cholesterol, mmol/L			
Median (IQR)	4.86 (4.10–5.70)	4.5 (3.7–5.2)	4.9 (4.1–5.8)
Missing data	49	7	42
Triglyceride, mmol/L			
Median (IQR)	1.02 (0.69–1.60)	0.95 (0.65–1.33)	1.04 (0.70–1.60)
Missing data	58	7	51
Creatinine			
Median (IQR)	76.0 (65.0–91.0)	89.0 (71.8–114.3)	75.0 (65.0–90.0)
eGFR			
Median (IQR)	90.9 (73.5–108.8)	74.6 (55.8–92.3)	92.1 (75.4–109.6)
Missing data	5	0	5
Systolic blood pressure			
Median (IQR)	129 (114–145)	129 (110–142)	129 (115–145)
Diastolic blood pressure			
Median (IQR)	75.0 (65.0–84.0)	72.0 (61.0–80.3)	75.0 (65.0–84.0)
Missing data	17	0	17
Renal failure (eGFR < 60), n (%)	214/1708 (12.5%)	43/144 (29.8%)	171/1564 (9.0%)

* one-year follow-up; Abbreviations: BMI—body mass index; SBP—Systolic blood pressure; DBP—diastolic blood pressure; MACE—major adverse cardiovascular events; IQR—interquartile range; MI—myocardial infarction; GRACE—Global Registry of Acute Coronary Events; Hs-CRP—high sensitivity C-reactive protein; Hs-cTnT—high-sensitivity troponin T; TMAO—trimethyl-amine-N-oxide; Anti-ApoA-I IgGs—autoantibodies against apolipoprotein A-I; OD—optical density; HDL—high-density lipoprotein; LDL—low-density lipoprotein; eGFR—estimated glomerular filtration rate.

**Table 2:** One-year cumulative major adverse cardiovascular events (MACE) incidence according to univariate analyses.

Predictors	Levels	N MACE/N Total	One-Year Cumulative Incidence of MACE (95% CI)	*p*-Value *
Age	26–54 y	17/450	3.8 (2.0–5.6)	<0.0001
	55–64 y	22/443	5.0 (3.0–7.0)	
	65–74 y	32/418	7.7 (5.1–10.3)	
	≥75 y	73/402	18.4 (14.5–22.1)	
BMI, kg/m^2^	20–24.9	49/554	9.0 (6.5–11.3)	0.1569
	25–29.9	50/767	6.6 (4.8–8.4)	
	30–34.9	26/283	9.3 (5.8–12.6)	
	≥35	10/86	12.0 (4.7–18.8)	
GRACE score	≤99	9/318	2.9 (1.0–4.7)	<0.0001
	100–119	25/453	5.6 (3.4–7.7)	
	120–139	29/456	6.4 (4.1–8.7)	
	140–159	37/303	12.3 (8.5–16.0)	
	≥160	44/183	24.3 (17.8–30.3)	
Gender	female	35/363	9.8 (6.6–12.8)	0.3095
	male	109/1350	8.2 (6.7–9.6)	
Diabetes history	no	97/1403	7.0 (5.6–8.3)	<0.0001
	yes	47/310	15.5 (11.3–19.4)	
MI history	No	113/1467	7.8 (6.4–9.2)	0.0124
	yes	31/246	12.9 (8.5–17.0)	
Hypertension history	no	44/720	6.2 (4.4–7.9)	0.0041
	yes	100/993	10.2 (8.3–12.1)	
Dyslipidemia history	no	61/680	9.1 (6.9–7.9)	0.4057
	yes	82/1031	8.0 (6.4–12.1)	
Valvular history	no	134/1680	8.1 (6.8–9.4)	<0.0001
	yes	10/33	30.3 (12.7–44.3)	
Current smoker	no	96/990	9.6 (7.7–11.4)	0.0251
	yes	44/690	6.5 (4.6–8.3)	
Hs-CRP, mg/L	0–0.99	12/299	4.1 (1.8–6.3)	<0.0001
	1–1.99	18/280	6.5 (3.5–9.3)	
	2–9.99	55/614	9.1 (6.8–11.4)	
	≥10	48/317	15.4 (11.3–19.4)	
Hs-cTnT, ng/L	0–0.14	40/647	6.3 (4.4–8.1)	0.0001
	0.15–0.52	29/379	7.8 (5.0–10.5)	
	>0.52	65/493	13.3 (10.3–16.3)	
TMAO, µmol/L	<2	25/340	7.4 (4.6–10.2)	0.0001
	2–2.99	12/350	3.5 (1.5–5.4)	
	3–3.99	13/206	6.4 (3.0–9.7)	
	4–6.99	27/257	10.6 (6.7–14.3)	
	≥7	29/200	14.7 (9.6–19.4)	

* Log-rank test. Abbreviations: BMI—body mass index; GRACE—Global Registry of Acute Coronary Events; MI—myocardial infarction; Hs-CRP—high sensitivity C-reactive protein; Hs-cTnT—high-sensitivity troponin T; TMAO—trimethyl-amine-N-oxide.

**Table 3:** One-year cumulative major adverse cardiovascular events (MACE) incidence according to univariate analyses.

Predictors	Levels	N MACE/N Total	One-Year Cumulative Incidence of MACE (95% CI)	*p*-Value *
Anti-ApoA-I IgGs, OD	≤0.45	31/442	7.1 (4.7–9.5)	0.0323
	0.46–0.65	24/396	6.1 (3.7–8.5)	
	0.66–0.95	48/425	11.5 (8.4–14.5)	
	>0.95	41/450	9.2 (6.5–11.9)	
HDL, mmol/L	<1	41/511	8.2 (5.7–10.5)	0.6210
	1–1.3	48/626	7.8 (5.6–9.8)	
	>1.3	47/511	9.3 (6.7–11.8)	
LDL, mmol/L	<2.5	59/492	12.3 (9.3–15.1)	0.0005
	2.5–3.5	46/580	8.0 (5.8–10.2)	
	>3.5	31/573	5.5 (3.6–7.3)	
Total cholesterol, mmol/L	<4.5	98/606	11.4 (8.8–13.9)	0.0027
	4.5–5.5	41/573	7.2 (5.1–9.3)	
	>5.5	28/485	5.9 (3.7–7.9)	
Triglyceride, mmol/L	<0.80	46/527	8.8 (6.3–11.2)	0.0653
	0.80–1.30	56/564	10.1 (7.6–12.6)	
	>1.30	35/564	6.3 (4.2–8.3)	
Creatinine, µmol/L	<70	31/606	5.2 (3.4–7.0)	<0.0001
	70–85	35/550	6.5 (4.4–8.5)	
	>85	78/557	14.2 (11.2–17.1)	
NT-proBNP, ng/L	<200	18/511	3.6 (1.9–5.2)	<0.0001
	200–1000	36/514	7.1 (4.8–9.3)	
	>1000	80/493	16.5 (13.1–19.7)	
SBP, mmHg	<120	47/561	8.4 (6.1–10.7)	0.5490
	120–140	60/649	9.4 (7.1–11.6)	
	>140	37/503	7.5 (5.2–9.8)	
DBP, mmHg	<70	59/602	9.9 (7.5–12.3)	0.2120
	70–80	49/565	8.8 (6.4–11.1)	
	>80	36/529	6.9 (4.7–9.0)	
eGFR <60 mL/min	no	101/1595	6.9 (5.6–8.1)	<0.0001
	yes	43/257	20.3 (14.7–25.6)	

* Log-rank test. Abbreviations: MACE—major adverse cardiovascular events; Anti-ApoA-I IgGs—autoantibodies against apolipoprotein A-I; OD—optical density; HDL—high-density lipoprotein; LDL—low-density lipoprotein; NT-proBNP—N-terminal pro-brain natriuretic peptide; SBP—Systolic blood pressure; DBP—diastolic blood pressure; eGFR—estimated glomerular filtration rate.

**Table 4:** Autoantibodies against apolipoprotein A-I (Anti-apoA-I IgGs) and major adverse cardiovascular events (MACE) (myocardial infarction, stroke, or cardiovascular (CV) death) risk according to Cox-regression analyses.

Predictors	Unadjusted HR (95% CI)	*p*-Value	Adjusted HR (95% CI) **	*p*-Value
Anti-ApoA-I IgGs OD				
≤0.45, Q1	Reference group	0.0338 *	Reference group	0.0496 *
0.46–0.65, Q2	0.85 (0.50–1.46)	0.5625	0.82 (0.48–1.40)	0.4682
0.66–0.95, Q3	1.64 (1.04–2.57)	0.0322	1.56 (0.99–2.44)	0.0558
>0.95, Q4	1.30 (0.82–2.08)	0.2657	1.23 (0.77–1.97)	0.3808
GRACE score 2.0				
≤99	Reference group	<0.0001 *	Reference group	<0.0001 *
100–119	1.96 (0.92–4.20)	0.0931	1.99 (0.93–4.26)	0.0772
120–139	2.27 (1.07–4.79)	0.0321	2.25 (1.07–4.76)	0.0332
140–159	4.44 (2.14–9.19)	<0.0001	4.44 (2.14–9.20)	<0.0001
≥160	9.44 (4.61–19.35)	<0.0001	9.36 (4.57–19.18)	<0.0001

* *p*-Value for the overall association between the factor (all levels) and risk of MACE; ** Adjusted for the GRACE score 2.0. Abbreviations: HR—hazard ratio; OD—optical density; GRACE—Global Registry of Acute Coronary Events.

**Table 5:** Exploring the factors associated with autoantibodies against apolipoprotein A-I (anti-apoA-I IgG) levels.

	Anti-apoA-I IgG Levels					
	All Patients	≤0.45 Q1	>0.45 and ≤0.65 Q2	>0.65 and ≤0.95 Q3	>0.95 Q4	*P* *
Age, years						
median (IQR)	64 (54–74)	63 (53–74)	63 (54–74)	64 (54–72)	65 (55–75)	0.1787
26–54 y	450 (26.3%)	122 (27.6%)	111 (28.0%)	109 (25.6%)	108 (24.0%)	
55–64 y	443 (25.9%)	121 (27.4%)	102 (25.8%)	114 (26.8%)	106 (23.6%)	
65–74 y	418 (24.4%)	99 (22.4%)	92 (23.2%)	110 (25.9%)	117 (26.0%)	
≥75 y	402 (23.5%)	100 (22.6%)	91 (23.0%)	92 (21.6%)	119 (26.4%)	
BMI, kg/m^2^						
median (IQR)	26.5 (24.2–29.4)	26.2 (24.2–29.3)	26.4 (24.2–29.3)	26.8 (24.3–29.7)	26.6 (24.2–29.4)	0.6434
20–24.9	554 (32.8%)	154 (35.2%)	128 (32.4%)	134 (32.2%)	138 (31.3%)	
25–29.9	767 (45.4%)	189 (43.2%)	187 (47.3%)	182 (43.8%)	209 (47.4%)	
30–34.9	283 (16.7%)	75 (17.1%)	62 (15.7%)	73 (17.5%)	73 (16.6%)	
≥35	86 (5.1%)	20 (4.6%)	18 (4.6%)	27 (6.5%)	21 (4.8%)	
missing data	23	4	1	9	9	
Female, n (%)	363/1713 (21.2%)	102/442 (23.1%)	87/396 (22.0%)	93/425 (21.9%)	81/450 (18.0%)	0.2669
Current smoker, n (%)	690/1680 (41.1%)	174/436 (39.9%)	147/390 (37.7%)	183/419 (43.7%)	186/435 (42.8%)	0.2875
Diabetes history, n (%)	310/1713 (18.1%)	97/442 (21.9%)	66/396 (16.7%)	83/425 (19.5%)	64/450 (14.2%)	0.0177
Hypertension history, n (%)	720/1713 (42.0%)	248/442 (56.1%)	234/396 (59.1%)	247/425 (58.1%)	264/450 (58.7%)	0.8191
MI history, n (%)	246/1713 (14.4)	53/442 (12.0%)	52/396 (13.1%)	73/425 (17.2%)	68/450 (15.1%)	0.1416
Cholesterolemia history, n (%)	1031/1711 (60.3%)	276/441 (62.6%)	247/395 (62.5%)	257/425 (60.5%)	251/450 (55.8%)	0.1311
Valvular history, n (%)	33/1713 (1.9%)	4/442 (0.9%)	6/396 (1.5%)	8/425 (1.9%)	15/450 (3.3%)	0.0572
GRACE score						
median (IQR)	123 (106–142)	123 (106–142)	123 (106–140)	123 (104–144)	123 (106–143)	0.7916
≤99	318 (18.6%)	83 (18.8%)	74 (18.7%)	84 (19.8%)	77 (17.1%)	
100–119	453 (26.4%)	121 (27.4%)	110 (27.8%)	100 (23.5%)	122 (27.1%)	
120–139	456 (26.6%)	115 (26.0%)	103 (26.0%)	115 (27.1%)	123 (27.3%)	
140–159	303 (17.7%)	84 (19.0%)	65 (16.4%)	76 (17.9%)	78 (17.3%)	
≥160	183 (10.7%)	39 (8.8%)	44 (11.1%)	50 (11.8%)	50 (11.1%)	
CRP, mg/L						
median (IQR)	3.0 (1.2–8.0)	2.60 (1.10–7.70)	2.70 (1.00–6.40)	3.30 (1.40–8.25)	3.10 (1.30–9.20)	0.1704
0–0.99	299 (19.8%)	77 (20.2%)	80 (22.7%)	63 (16.6%)	79 (19.9%)	
1–1.99	280 (18.5%)	76 (19.9%)	67 (19.0%)	71 (18.7%)	66 (16.6%)	
2–9.99	614 (40.7%)	149 (39.1%)	141 (39.9%)	166 (43.8%)	158 (39.8%)	
≥10	317 (21.0%)	79 (20.7%)	65 (18.4%)	79 (20.8%)	94 (23.7%)	
missing data	203	61	43	46	53	
Hs-cTnT, ng/L						
median (IQR)	0.22 (0.07–0.75)	0.20 (0.06–0.67)	0.21 (0.06–0.66)	0.24 (0.07–0.87)	0.26 (0.07–0.83)	0.1947
0–0.14	647 (42.6%)	172 (45.0%)	155 (43.7%)	158 (41.1%)	162 (40.7%)	
0.15–0.52	379 (25.0%)	98 (25.7%)	86 (24.2%)	93 (24.2%)	102 (25.6%)	
>0.52	493 (32.5%)	112 (29.3%)	114 (32.1%)	133 (34.6%)	134 (33.7%)	
missing data	194	60	41	41	52	
TMAO						
median (IQR)	2.93 (1.99–4.97)	2.85 (1.89–4.97)	2.96 (2.01–4.98)	2.93 (2.00–4.46)	3.05 (2.07–5.43)	0.4744
<2	340 (25.1%)	98 (28.3%)	74 (23.9%)	83 (25.1%)	85 (23.2%)	
2–2.99	350 (25.9%)	87 (25.1%)	84 (27.2%)	86 (26.0%)	93 (25.3%)	
3–3.99	206 (15.2%)	42 (12.1%)	51 (16.5%)	60 (18.1%)	53 (14.4%)	
4–6.99	257 (19.0%)	66 (19.1%)	50 (16.2%)	63 (19.0%)	78 (21.3%)	
≥7	200 (14.8%)	53 (15.3%)	50 (16.2%)	39 (11.8%)	58 (15.8%)	
missing data	360	96	87	94	83	

* Kruskal-Wallis or Chi-2 tests (Fisher exact test for liver history). Abbreviations: IQR—interquartile range; BMI—body mass index; MI—myocardial infarction; GRACE—Global Registry of Acute Coronary Events; CRP—C-reactive protein; hs-cTnT—high-sensitivity troponin T; TMAO—trimethyl-amine-N-oxide.

**Table 6:** Exploring the factors associated with autoantibodies against apolipoprotein A-I (anti-apoA-I IgG) levels.

	Anti-apoA-I IgG Levels					
	All Patients	≤0.45 Q1	>0.45 and ≤0.65 Q2	>0.65 and ≤0.95 Q3	>0.95 Q4	*P* *
SBP, mmHg						
Median (IQR)	129 (114–145)	129 (115–145)	129 (115–144)	128 (112–145)	130 (114–146)	0.9714
Missing data	0	0	0	0	0	
DBP, mmHg						
Median (IQR)	75 (65–84)	75 (66–84)	75 (65–84)	73 (65–84)	74 (63–84)	0.6476
Missing data	17	7	2	4	4	
Hemoglobin, g/L						
Median (IQR)	138 (127–149)	137 (124–149)	138 (126–148)	139 (129–148)	139 (128–150)	0.3715
Missing data	87	30	19	19	19	
Hematocrit, %						
Median (IQR)	41 (38–43.3)	41 (37–43)	40 (37–43)	41 (38–44)	41 (38–44)	0.2161
Missing data	76	26	14	18	18	
Leucocytes, G/L						
Median (IQR)	9.6 (7.4–12.1)	9.5 (7.3–12.0)	9.2 (7.1–12.0)	9.8 (7.9–12.1)	9.8 (7.6–12.3)	0.0340
Missing data	73	25	14	17	17	
Erythrocytes, T/L						
Median (IQR)	4.5 (4.1–4.8)	4.5 (4.1–4.9)	4.4 (4.1–4.8)	4.5 (4.2–4.8)	4.5 (4.1–4.8)	0.3240
Missing data	84	28	17	19	20	
Lymphocytes, %						
Median (IQR)	1.62 (1.16–2.35)	1.67 (1.20–2.55)	1.54 (1.09–2.31)	1.69 (1.19–2.34)	1.58 (1.11–2.22)	0.0526
Missing data	395	118	95	87	95	
Neutrophils, G/L						
Median (IQR)	7.3 (5.3 to 10.0)	7.0 (5.0 to 9.7)	7.2 (4.7 to 10.1)	7.4 (5.5 to 9.9)	7.6 (5.5 to 10.1)	0.1500
Missing data	412	122	97	93	100	
Monocytes, %						
Median (IQR)	0.62 (0.45 to 0.95)	0.63 (0.45 to 1.05)	0.56 (0.41 to 0.92)	0.64 (0.48 to 0.99)	0.61 (0.45 to 0.90)	0.1220
Missing data	398	118	96	88	96	
Basophils, %						
Median (IQR)	0.03 (0.01–0.07)	0.03 (0.02–0.08)	0.03 (0.01–0.07)	0.03 (0.01–0.07)	0.03 (0.01–0.06)	0.7491
Missing data	399	118	95	88	98	
Eosinophils, %						
Median (IQR)	0.08 (0.02–0.19)	0.10 (0.03–0.21)	0.08 80.03–0.20)	0.07 (0.02–0.17)	0.07 (0.02–0.16)	0.0237
Missing data	401	119	96	88	98	
Thromboctyes, G/L						
Median (IQR)	215 (183–257)	226 (188–272)	211 (179–250)	218 (185–253)	208 (176–255)	0.0026
Missing data	85	27	15	21	22	
Total cholesterol, mmol/L						
Median (IQR)	4.9 (4.1–5.7)	4.9 (4.1–5.8)	4.9 (4.0–5.8)	4.9 (4.1–5.7)	4.7 (4.0–5.6)	0.1602
Missing data	49	13	13	11	12	
HDL, mmol/L						
Median (IQR)	1.13 (0.69–1.60)	1.14 (0.93–1.40)	1.14 (0.96–1.40)	1.13 (0.94–1.40)	1.10 (0.93–1.33)	0.2852
Missing data	65	20	15	14	16	
LDL, mmol/L						
Median (IQR)	308 (2.34–3.84)	3.07 (2.32–3.94)	3.13 (2.40–3.82)	3.08 (2.37–3.81)	2.99 (2.26–3.81)	0.7418
Missing data	68	20	16	16	16	
Triglyceride						
Median (IQR)	1.02 (0.69–1.60)	1.08 (0.72–1.70)	1.05 (0.69–1.68)	1.00 (0.66–1.51)	0.99 (0.69–1.48)	0.2165
Missing data	58	17	15	13	13	
Creatinine, µmol/L						
Median (IQR)	76 (65–91)	75 (64–88)	76 (66–91)	76 (65–92)	77 (66–92)	0.3484
Missing data	0	0	0	0	0	
NT-ProBNP, ng/L						
Median (IQR)	414 (131–1436)	386 (118–1462)	355 (129–1196)	433 (132–1627)	544 (159–1633)	0.0390
Missing data	195	60	41	42	52	
eGFR, mL/min						
Median (IQR)	90.9 (73.5–108.8)	92.1 (75.8–110.9)	91.0 (73.5–107.2)	89.8 (73.5–108.3)	90.7 (70.5–108.6)	0.4791
Missing data	5	1	1	2	1	
Renal failure (eGFR <60), n (%)	214/1708	47/441	53/395	56/423	58/449	0.5828

* Kruskal-Wallis or Chi-2 tests (Fisher exact test for liver history). Abbreviations: Anti-apoA-I IgG—autoantibodies against apolipoprotein A-I; SBP—systolic blood pressure; DBP—diastolic blood pressure; IQR—interquartile range; HDL—high-density lipoprotein; LDL—low-density lipoprotein; NT-ProBNP—N-terminal pro-brain natriuretic peptide; eGFR—estimated glomerular filtration rate.

## Supplementary Materials

The following are available online at https://www.mdpi.com/2077-0383/8/7/1002/s1, Figure S1: Hazard ratio for the association between anti-apoA-I IgGs levels and specific endpoints of the composite MACE outcome assessed with Cox models accounting for the potential non linearity (with splines) of the relationship and adjusted for categories of the GRACE score, Table S1: Spearman Correlation coefficients between anti-apoA-I IgG and other continuous variables of interest.
