# Supplementary material for: Non-Linear Relationship between Anti-Apolipoprotein A-1 IgGs and Cardiovascular Outcomes in Patients with Acute Coronary Syndromes
Source: J Clin Med. 2019 Jul 9;8(7):1002. doi: 10.3390/jcm8071002 (PMC6679072; doi:10.3390/jcm8071002)
Supplement: Supplementary file 1 [file jcm-08-01002-s001.pdf]

Figure S1: Hazard ratio for the association between anti-apoA-1 IgG levels and specific endpoints of the composite MACE outcome assessed with Cox models accounting for the potential non linearity (with splines) of the relationship and adjusted for categories of the GRACE score.

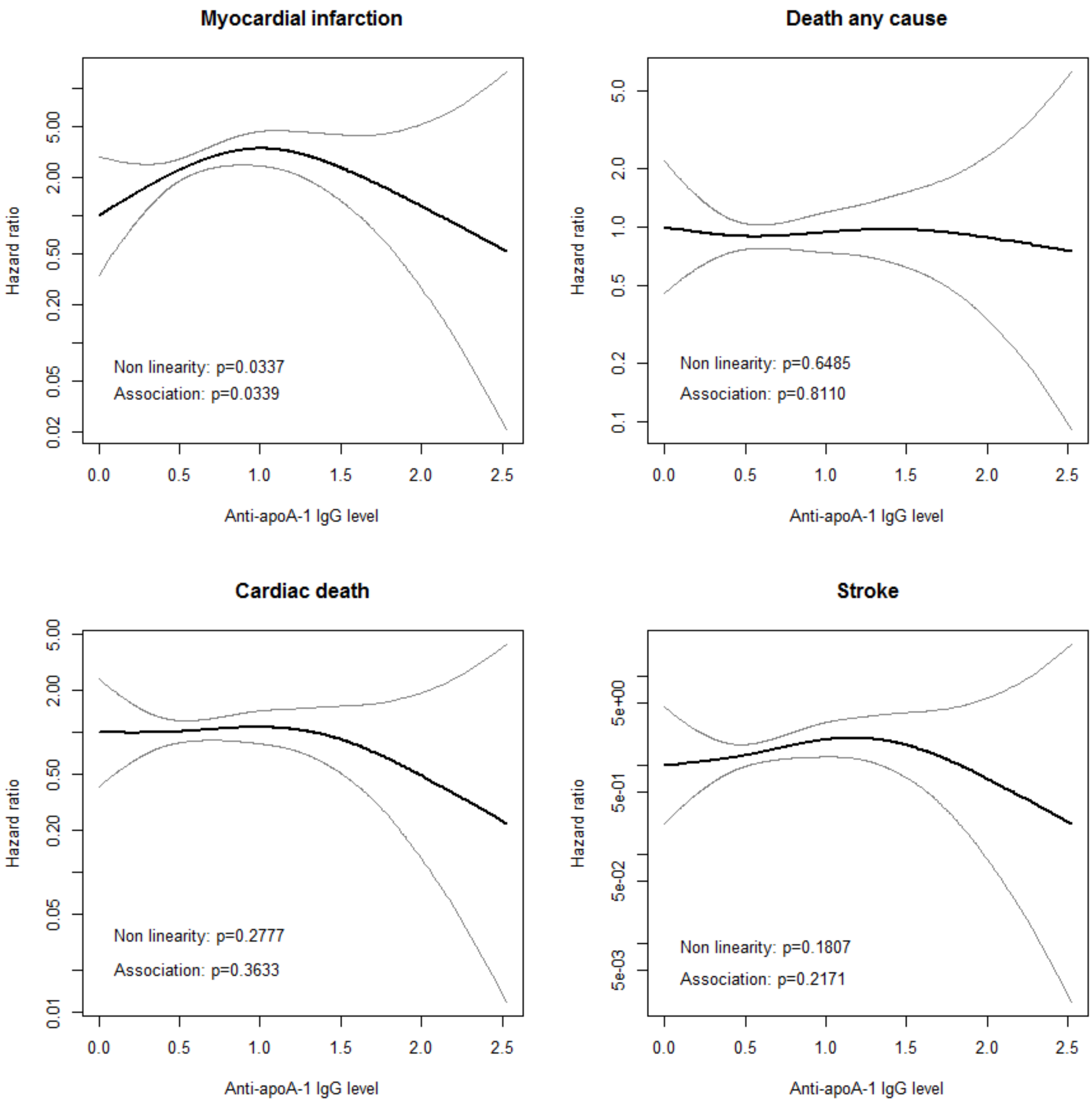

Table S1: Spearman Correlation coefficients between anti-apoA-1 IgG and other continuous variables of interest

Pairs of Variables	Spearman correlation coefficient	P value
Anti-ApoA-1 IgG & TMAO	0.032	0.23
Anti-ApoA-1 IgG & Hs-CRP	0.038	0.14
Anti-ApoA-1 IgG & Hs-cTnT	0.042	0.10
Anti-ApoA-1 IgG & NT-proBNP	0.061	0.02
Anti-ApoA-1 IgG & Age	0.046	0.06
Anti-ApoA-1 IgG & BMI	0.023	0.35
Anti-ApoA-1 IgG & SBP	0.014	0.56
Anti-ApoA-1 IgG & DBP	-0.017	0.48
Anti-ApoA-1 IgG & Haemoglobin	0.041	0.09
Anti-ApoA-1 IgG & Hematocrit	0.048	0.052
Anti-ApoA-1 IgG & Leucocytes	0.046	0.06
Anti-ApoA-1 IgG & Erythrocytes	0.032	0.20
Anti-ApoA-1 IgG & Lymphocytes	-0.037	0.18
Anti-ApoA-1 IgG & Neutrophils	0.058	0.038
Anti-ApoA-1 IgG & Monocytes	-0.018	0.52
Anti-ApoA-1 IgG & Basophils	-0.010	0.71
Anti-ApoA-1 IgG & Eosinophils	-0.071	0.01
Anti-ApoA-1 IgG & Thrombocytes	-0.071	0.004
Anti-ApoA-1 IgG & Total cholesterol	-0.045	0.07
Anti-ApoA-1 IgG & HDL-C	-0.030	0.22
Anti-ApoA-1 IgG & LDL-C	-0.023	0.35
Anti-ApoA-1 IgG & Triglycerides	-0.043	0.08
Anti-ApoA-1 IgG & Creatinine	0.046	0.06
Anti-ApoA-1 IgG & GRACE score 2.0	0.021	0.37
